# Specific binding of a polymer chain to a sequence of surface receptors

**DOI:** 10.1038/s41598-017-17581-x

**Published:** 2017-12-08

**Authors:** Samuel Bell, Eugene M. Terentjev

**Affiliations:** Cavendish Laboratory, University of Cambridge, Cambridge, CB3 0HE UK

## Abstract

This paper considers a biologically relevant question of a Gaussian chain (such as an unfolded protein) binding to a sequence of receptors with matching multiple ligands distributed along the chain. Using the characteristic time for a tethered ligand to bind to a surface receptor, we study the case of multiple binding to a linear sequence of receptors on the surface. The tethered binding time is determined by the entropic barrier for the chain to be stretched sufficiently to reach the distant receptor target, and a restriction on chain conformations near the substrate. Adsorption (multiple-site binding) is shown to be dominated by a simple zipper sequence, only occasionally accelerated by loop formation. However, when the number of receptors increases, a competing rate-limiting process takes over: the center of mass of the remaining free chain has to drift down the line of receptors, which takes longer when the receptors are close and the entropic pulling force is low. As a result, the time for the complete chain adsorption is minimised by a certain optimal number of receptors, depending on the distance to be traversed by the free end, and the chain length.

## Introduction

Ordered self-assembly relies on the organisation and binding of long-chain macromolecules into a coherent structure. In biology, this is achieved through the use of specific interactions, matching ligands, and distinct binding sites. The kinetics of these processes is a broad and rich topic, which offers fundamental insight into the construction of structured and functional aggregates. Surface adsorption of a polymer is not a new topic; industrial interest in colloid stabilisation and oil extraction led the early theoretical pioneers – Silberberg^1^, de Gennes^2,3^ and Alexander^4 ^– to study the equilibrium properties of polymer chains close to surfaces.

As polymer physics matured, there grew a need to understand the kinetics of adsorption. Existing work distinguishes between two regimes, depending on the free energy barrier presented to monomers binding on the surface: chemisorption if the reaction barrier is high, and physisorption if the barrier is low and the characteristic time to establish a bond on contact is short. In chemisorption, the reaction time of the monomers is larger than the time for the polymer to return to an equilibrated state, so the process becomes quasistatic. Theory^5,6^, experiment^7^, and simulations^6–10^, have all shown that two mechanisms control chemisorption: the zipping down of sequential monomers, and the formation of extra nucleation points via loop formation. Loop formation lowers the adsorption time relative to a simple sequential zipping mechanism, and so chemisorption is said to take place via the accelerated zipping mechanism.

Physisorption is, on average, a simple zipping mechanism: sequential monomers very quickly attach to the surface, leaving no time for loop formation^6,10–12^. This forces the chain out of equilibrium, as the remaining unabsorbed segment initially moves more slowly than the chain zipping down. The precise scaling of this adsorption time depends on the strength of the polymer-surface attraction^6^. For irreversible physisorption, the intermediate chain conformation combining a stretched tether at the zipping end, and a coil at the free end, is known as the ‘stem and flower’ model, and was first described by Brochard-Wyart^13^ for tethered polymers under strong shear flow.

Many of the systems considered are entirely homogeneous polymer chains with no specificity of binding sites, while others examined copolymers attaching to uniform surfaces^9,14^. Li *et al*. have studied stripe-patterned surfaces^15^, and copolymers of one attractive and one inert monomer type^16^, but many processes in self-assembly are much more specific than this. These processes, such as DNA hairpin formation^17,18^, still show zipper kinetics.

In a study of cell adhesion, Jeppesen *et al*.^19,20^ had this problem for one specific binding site, where ligands tethered to the cell surface by flexible chains could also associate with the matching receptor on the adjacent cell. They found a dependence on the configuration of the polymer tether: in particular, how often the chains entered extended configurations to reach the distant receptors. Their treatment did not extend to an analytical expression of the binding rate, or multiple binding sites. Tethered binding is a process found in the thrombin receptor^21–23^, and in cytoskeletal molecular motors (dynein, kinesin and myosin)^24,25^, which all involve a ligand at the end of a flexible ‘arm’ looking for a site on a substrate a certain distance away.

**Figure 1:**
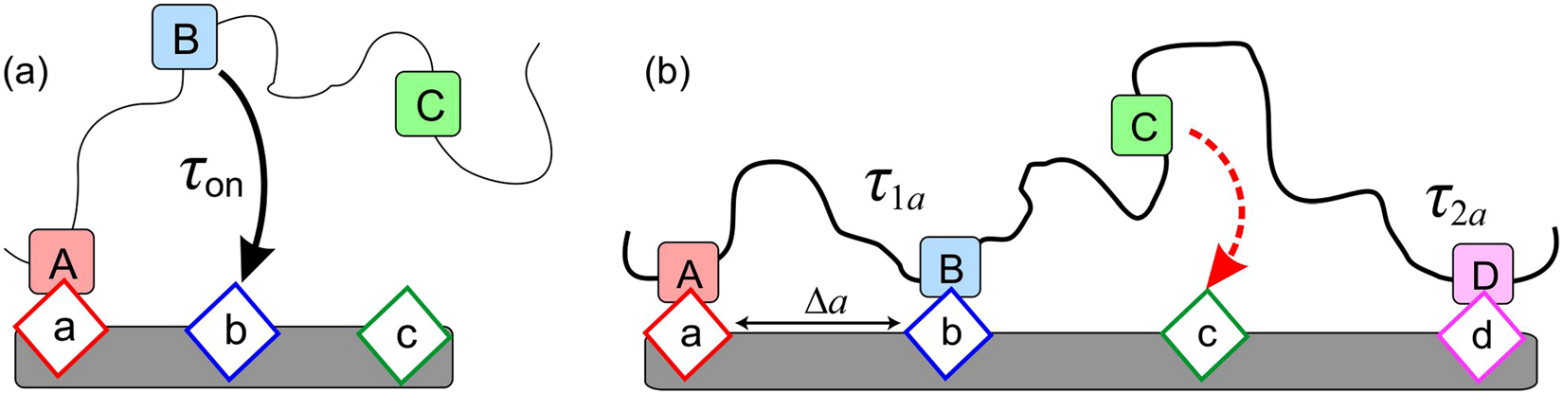
A scheme of ordered self-assembly of a polymer chain binding to a specific sequence of receptors on a substrate. (**a**) The single binding time *τ*_on_ is a function of chain length A-B and the receptor separation a-b. (**b**) Each surface receptor is separated by a distance Δ*a*. In this scenario, the ligand [B] has bound via a single step, with typical time *τ*_1*a*_ = *τ*_on_(Δ*a*), and the ligand [D] has bound with a double step of time *τ*_2*a*_; the ligand [C] will then bind quickly.

Our problem, therefore, consists of two aspects: using the knowledge of the time required for the chain to ‘reach for a distant target’ (which depends on the parameters of the tether chain and the receptor) – see Fig. 1(a), we first evaluate the average binding time to a sequence of such receptors, Fig. 1(b). This process of chain adsorption may proceed via different pathways, involving purely sequential (zipper) single steps, or multiple-distance looping events. It turns out that, for sufficiently separated binding sites, the simple zipping mechanism becomes the preferred pathway. Secondly, we consider the ‘stem and flower’ effect, and show that for a chain looking to bind its free end to a target a distance *a* away as fast as possible, non-equilibrium effects associated with a slow drift of the chain towards the receptor targets defines a certain optimal number of intermediate receptors that achieves the fastest mean adsorption time.

## Accelerated zipper binding

Consider an ideal polymer chain of *N* segments of length *b*, which is grafted at the origin to a flat surface, where the last (*N*th) monomer is the binding ligand. Its matching receptor is located on the same surface, a distance *a* away, Fig. 1(a). The effect of the reflecting surface on polymer chain configurations has a very long history^26^, culminating with the classical work of Edwards and Freed^27^ about the ‘chain in a box’. A number of excellent analytical theories have addressed the kinetics of polymer reactions, such as binding to a site or to itself, forming a ring^28,29^. In our case, the difference is that the first monomer is constrained by grafting while the end of the chain is searching for the distant target. One can construct an effective radial probability distribution for the distance *ρ* from the target receptor ***r***_*N*_ to the binding site^30^:

1 $${P}_{eq}(\rho )\approx \frac{2b}{a}\sqrt{\frac{N\pi }{6}}{(\frac{3}{2\pi N{b}^{2}})}^{\mathrm{3/2}}{e}^{-\frac{\mathrm{3(}{a}^{2}+{\rho }^{2})}{2N{b}^{2}}}{I}_{1}(\frac{3a\rho }{N{b}^{2}}),$$

Where *I*_1_(*x*) is a modified Bessel function of the first kind. That radial probability distribution *P*_*eq*_(*ρ*) is amenable to the mean first passage time approach of Szabo *et al*.^28^

2 $${\tau }_{{\rm{on}}}=2\pi {\int }_{\varepsilon }^{\infty }d\rho {[D{\rho }^{2}{P}_{eq}(\rho )]}^{-1}{[{\int }_{\rho }^{\infty }d\rho ^{\prime} {\rho ^{\prime} }^{2}{P}_{eq}(\rho ^{\prime} )]}^{2}\approx \frac{{N}^{2}{b}^{4}}{9D{\varepsilon }^{2}}{e}^{3{a}^{2}\mathrm{/2}N{b}^{2}}\mathrm{.}$$

where we assume the receptor zone is hemispherical, with a small radius *ε*, and the diffusion coefficient of the free end of the chain, *D* = *k*_*B*_ *T*/*γ*, is a constant equal to the diffusion coefficient of a single monomer in solution. Note the effect of the entropic barrier in the ‘thermal activation’ exponential factor: to reach the target, the chain has to raise its free energy to Δ*G* = 3*k*_*B*_ *Ta* ^2^/2*Nb* ^2^. Also note that the mean first time to reach the surface receptor diverges as the reaction volume *ε* → 0; the scaling in Eq. (2) is enhanced by the exclusion boundary condition for the chain segment to get anywhere near the wall.

We can now use the expression (2) for *τ*_on_(*a*,*N*) to examine a simplified version of the multiple-site binding problem. Let us consider *M* binding sites spaced evenly along a polymer chain of total length *N* (so that the ligands are Δ*N* = *N/M* monomers apart, along the chain). As in the single-site problem, we take the first segment of the chain to be already bound (grafted) to the surface, so there are *M* binding events yet to occur in total. The receptors for these ligands are spaced at equal distances Δ*a* apart in a straight line on a plane reflecting surface, see Fig. 1(b). While it should not be difficult to consider arbitrary positioning of chain binding sites and surface receptors, we use this simplified geometry in the hope of finding a clear analytical result for the average binding time.

Assuming that each binding ligand on the chain associates with a specific receptor, the chain may form a loop by binding across several receptors, a distance *q*Δ*a* from the grafting point, see Fig. 1(b) for a *q* = 2 loop. The time to bind to a receptor a distance *q*Δ*a* away is

3 $${\tau }_{qa}=\frac{{(qN/M)}^{2}{b}^{4}}{9D{\varepsilon }^{2}}{e}^{\frac{\mathrm{3(}q{\rm{\Delta }}a{)}^{2}M}{2qN{b}^{2}}}={q}^{2}{e}^{\frac{\mathrm{3(}q-\mathrm{1)}M{\rm{\Delta }}{a}^{2}}{2N{b}^{2}}}\cdot {\tau }_{1a},$$

where the single-step binding time *τ*_1*a*_ = *τ*_on_(Δ*a*,*N*/*M*) from Eq. (2). If the chain does not bind sequentially, but with a loop forming (for example, next nearest binding in Fig. 1(b)), the subsequent binding of the ‘middle’ ligands is much faster (site [C] between [B] and [D] in Fig. 1(b)), and therefore is not a rate-limiting step. The combination of single and multiple steps is the accelerated zipper mechanism.

The kinetics of both single and multiple steps can be understood as a continuous-time Markov chain^31,32^, with *M* + 1 discrete states corresponding to how far along the chain the final binding event has been. This means we can write the rate equations in vector form: d*P*/d*t* = *Q* ⋅ *P*, where *Q* is known as the rate matrix. This is called the backward Kolmogorov equation. The (*M* + 1) × (*M* + 1) rate matrix has the following form:

4 $$Q=(\begin{array}{cccccc}-\sum _{q=1}^{M}{k}_{q} & {k}_{1} & {k}_{2} & {k}_{3} & ... & {k}_{M}\\ 0 & -\sum _{q=1}^{M-1}{k}_{q} & {k}_{1} & {k}_{2} & ... & {k}_{M-1}\\ 0 & 0 & \ddots  & \ddots  & \ddots  & \vdots \\ \vdots  & ... & 0 & -\sum _{q=1}^{2}{k}_{q} & {k}_{1} & {k}_{2}\\ \vdots  & 0 & ... & 0 & -{k}_{1} & {k}_{1}\\ 0 & 0 & 0 & ... & 0 & 0\end{array})$$

where *k*_*q*_ = 1/*τ*_*qa*_ are the rates of binding to a receptor a distance *q*Δ*a* away. Instead of explicitly solving the Kolmogorov equation, we can rely on the following fundamental result to derive the recursive relations for mean first passage times 〈*τ*(*M* + 1 − *i*)〉 from state *i* to the final fully bound state (across *M* + 1 − *i* receptors, where state *i* = 0 is the tethered chain with no receptors bound, and *i* = *M* + 1 is when the final *M*th receptor is bound)^32^:

5 $$\sum _{j}{Q}_{ij}\langle \tau (M+1-j)\rangle =-\mathrm{1,}$$

for all states 1 ≤ *i* < *M* + 1. If we start in the final absorbing state, then the mean first passage time is zero by definition, and so 〈*τ*(0)〉 = 0. The remaining 〈*τ*(*i*)〉 can then be constructed recursively.

**Figure 2:**
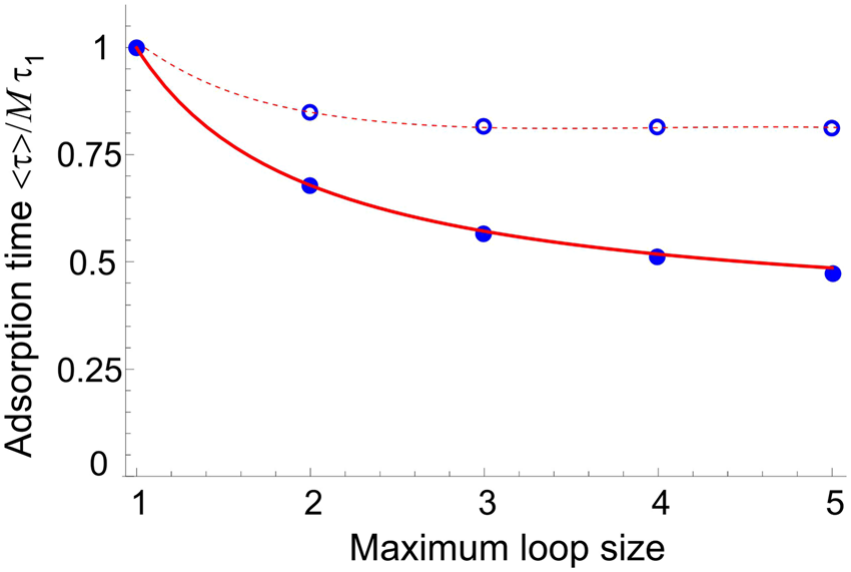
The plot of the (scaled) average adsorption time 〈*τ*(*M*)〉 for *M* = 20, when the multiple-step jumps (loops) are allowed. The *x*-axis indicates the largest jump allowed to accelerate the zipper binding. Filled symbols • represent the maximum effect, when the exponential entropic-penalty factor in *τ*_1*a*_ is equal to one (i.e. the distance Δ*a* = 0); the open symbols ○ represent the reduced acceleration effect when Δ*a* ^2^ = Δ*Nb* ^2^: already an almost negligible deviation from a simple zipper time past the loop size = 2.

While Eq. (5) is a full solution to the problem of mean binding time, it does not indicate how important loop formation is in the binding process. To make progress, we compare the binding times *τ*_*qa*_. For multiple steps, the rapidly increasing entropic barrier to reach receptors further away means that the expected time for binding gets longer and longer. Figure 2 shows how the mean binding time across *M* = 20 receptors reduces as we include the possibility of longer jumps. It is clear that we are free to neglect steps past *q* = 2 when Δ*a* ^2^ ≥ Δ*Nb* ^2^ (the open symbols in Fig. 2, as *τ*_*qa*_ rapidly increases with *q*, and the adsorption time seems to rapidly approach a limiting value. For very closely spaced receptors ($\Delta {a}^{2}\ll (N/M){b}^{2}$) the ratio of *τ*_*qa*_/*τ*_1*a*_ = *q* ^2^, and Fig. 2 shows that although the largest reduction in binding time comes from increasing the maximum step to *q* = 2, larger size loops do still play an appreciable role. To offer a quantitative idea of how big a role they play, we manually fitted a curve to the closely spaced receptors in Fig. 2 (the solid red line), and found that the deviation from a fitted limiting value 〈*τ*_∞_〉 was 〈*τ*_*q*_〉 − 〈*τ*_∞_〉 ∝ 1/*q*.

In the regime Δ*a* ^2^ ≥ Δ*Nb* ^2^, we restrict the binding process to either binding at the closest available site, which takes an average time *τ*_1*a*_ and follows in a zipper sequence, or at the next nearest site, which takes *τ*_2*a*_, as shown in Fig. 1(b); all other binding events across greater distances are neglected (*k*_*i*_ = 0 for *i* > 2), see Fig. 2. Then, Eq. (5) defines a recurrence relation for arbitrary *M*:

6 $$\begin{array}{ccc}\langle \tau (1)\rangle  & = & \frac{1}{{k}_{1}},\quad \langle \tau (2)\rangle =\frac{2}{{k}_{1}+{k}_{2}},\\ ({k}_{1}+{k}_{2})\langle \tau (M)\rangle  & = & 1+{k}_{1}\langle \tau (M-1)\rangle +{k}_{2}\langle \tau (M-2)\rangle \quad \quad {\rm{f}}{\rm{o}}{\rm{r}}\quad M\ge 2.\end{array}$$

Using a standard generating function method to solve this recurrence relation^33^, we find that

7 $$\langle \tau (M)\rangle =\frac{M{k}_{1}^{2}+\mathrm{2(}M+\mathrm{1)}{k}_{1}{k}_{2}+2{k}_{2}^{2}(1+{(-\frac{{k}_{2}}{{k}_{1}+{k}_{2}})}^{M-1})}{{k}_{1}{({k}_{1}+2{k}_{2})}^{2}}\mathrm{.}$$

Since this expression is only valid for sufficient spacing, ${k}_{2}\ll {k}_{1}$, we are free to Taylor expand Eq. (7):

8 $$\langle \tau \mathrm{(1)}\rangle \approx M{\tau }_{1a}-\frac{\mathrm{2(}M-\mathrm{1)}{\tau }_{1a}^{2}}{{\tau }_{2a}}=(M-\frac{M-1}{2}{e}^{-\frac{3M{\rm{\Delta }}{a}^{2}}{2N{b}^{2}}}){\tau }_{1a}\mathrm{.}$$

**Figure 3:**
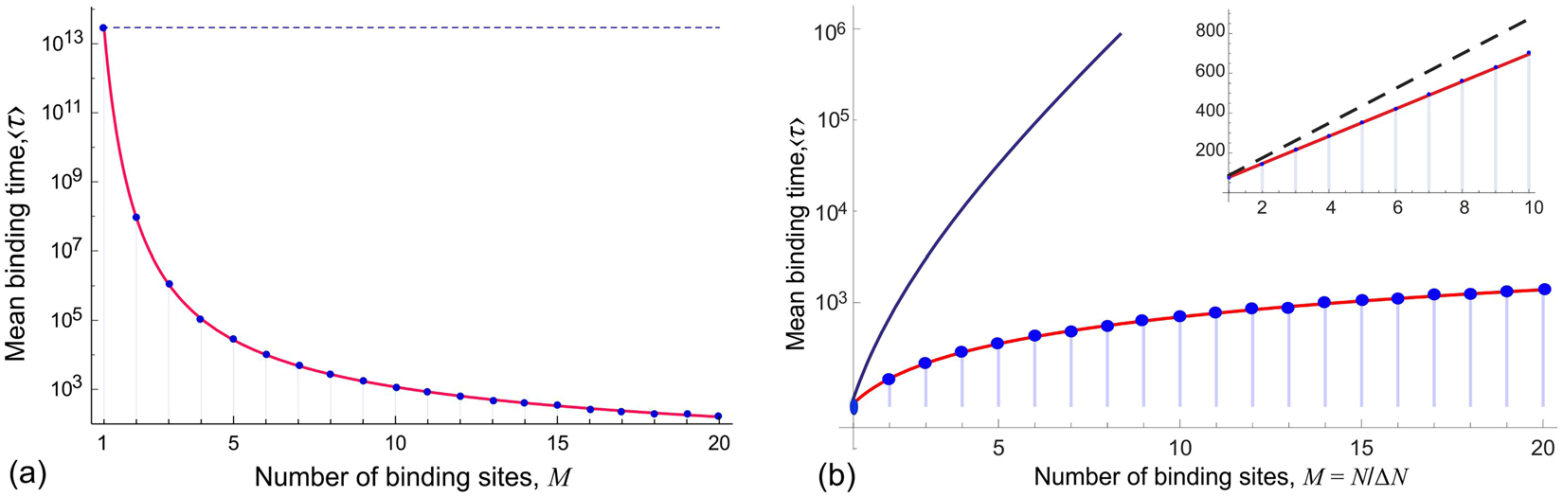
The mean time (in units of *b* ^2^/*D*, logarithmic scale) to bind the chain to the surface, as a function of the number *M* of equidistant binding sites. (**a**) A fixed chain *N* = 100 segments. The dashed blue line marks the case of *M* = 1, when only the *N*th segment has a binding ligand, reaching for a receptor *a* = 40*b* away. As the number of binding sites along the chain increases, the time to bind the final receptor dramatically reduces. (**b**) A fixed interval between receptors Δ*N* = 20, so *N* = *M*Δ*N*. The plot compares a chain with single binding site at a distance *a* = 3*Nb*/20 (solid blue line), and a chain with *M* = *N*/Δ*N* binding sites every 20 monomers, whose receptors are spaced at Δ*a* = *a*/*M* = 3*b*. In both cases, the end of the chain binds at the distance *a*. The inset illustrates that the typical binding time increases almost linearly with chain length or number of sites, in contrast to the exponential increase of this time for the single-site chain. The dashed black line indicates the line *Mτ*_1*a*_, which is the strictly single-step zipper binding pathway. The possibility of occasional double steps lowers the binding time of an ‘accelerated zipper’. In both plots, the blue dots represent the exact expression for 〈*τ*〉; the continuous red line is the plot of (8), where Δ*a* = *a*/*M*.

This expression is the first main result of our paper. Figure 3 shows the comparison of the approximation presented in Eq. (8), and the exact sum in Eq. (7), the latter plotted as discrete points at integer values of *M*. Evidently, the approximation (8) is virtually indistinguishable from the exact average binding time, when the probability of making a double step is small.

How does adding more binding sites along a chain length influence its time to bind to a surface? Let us consider a chain of fixed length *N*, as usual grafted at the origin. There is a binding ligand on the end of the chain, and Eq. (2) gives the mean time for it to bind at a receptor on the surface a long distance *a* away: *τ*_on_(*N*,*a*). Let us now add several more binding ligands on the chain, such that they have *N*/*M* monomers in between, and the matching sequence of equidistant receptor sites on the surface, such that they are a distance Δ*a* = *a*/*M* apart. The resulting decrease in binding time is plotted in Fig. 3(a). Note that the binding time is plotted on a logarithmic scale, so the effective decrease is quite dramatic when more binding sites are added to the chain. Equation (8) gives the scaling 〈*τ*〉 ∝ *M* ^−1^exp[*α*/*M*].

We also examine the situation where the binding site density is kept constant, i.e binding sites on the chain are equally spaced, and the matching receptors on the surface are always spaced the same distance Δ*a* apart, but vary the total length of the chain. In this case the total chain length *N* = *M*Δ*N*, and the distance to the last receptor is *a* = *M*Δ*a*. The results are plotted in Fig. 3(b) for the receptor density Δ*a* = 3Δ*Nb*/10 = 6*b*. The comparison is made with the mean binding time for the chain with only one binding site at the end, with the chain length and the distance to the single receptor related in the same way: *a* = 3*Nb*/10 away, to illustrate the role of overall chain length. This time increases almost exponentially, see Eq. (2) giving *τ*_on_ ∝ *N* ^2^exp[*αN*]. In contrast, the mean time to bind a sequence of receptors increases only ∼linearly with the chain length, illustrating that multiple sites massively enhance the binding rate. Note that a non-zero probability to make occasional double steps increases the binding rate even further, comparing with the straight zipper sequence, making it an ‘accelerated zipper’ process – this is illustrated by the linear plot in the inset of Fig. 3(b).

## Drift of center of mass

When we consider a single binding event, we do not consider the entire chain’s length, but instead just the length between the tether and the binding site. However, in a sequence of binding events, we must consider how the rest of the chain moves around at the time of binding. In a typical monomer-by-monomer physisorption of a chain to a uniform surface, the rapid binding of monomers to the surface moves the effective current grafting point away from the center of mass of the remaining free chain, resulting in the ‘stem and flower’ configuration of Brochard-Wyart^12,13^. This has an effect on the adsorption kinetics. Is there a similar effect when there is a section of chain between binding sites?

According to equilibrium polymer statistics, at the moment of a binding event, we would expect the remainder of the chain to be centered directly above the new grafting site. This stems from the Markovian treatment of the polymer chain, and is easily derived through Gaussian propagators. However, a study by Guérin *et al*.^29^ found that in reality, non-Markovian effects (i.e. non-Gaussian chain statistics before equilibrium is reached) play a large role in determining the dynamics of polymer configurations for reactions to a target in free space. The delay in reaching the equilibrium is often quite extended, so that the chain leaves the center of mass behind while reaching for a new target. This becomes clear if we consider the Rouse modes of the chain: the typical time for a monomer to fluctuate (travel a distance Δ*a*) is much smaller than that for the chain center of mass to do the same. As such, the polymer chain is ‘left behind’ when the next binding site finds its receptor, and we will need to consider the subsequent drift of the chain center of mass to the new grafting point. In this case, in contrast to the monomer-by-monomer physisorption, the rare binding steps result in a high stretching of the ‘stem’ and a relatively high force pulling the remaining free chain towards the new equilibrium around the new grafting site. The mean time to diffuse a distance for the center of mass to diffuse the distance Δ*a* is:

9 $${\tau }_{{\rm{c}}{\rm{o}}{\rm{m}}}=\frac{{N}_{f}{{\rm{\Delta }}a}^{2}}{D},$$

where *N*_*f*_ is the number of monomers in the remaining free chain, so that *N*_*f*_ *γ* is the effective friction constant for the free chain center of mass. As before, *D* = k_*B*_ *T*/*γ* is the diffusion constant for a single monomer. In order for the Szabo-based^28^ expression for the binding time *τ*_1*a*_ to be valid, we should have a chain in equilibrium configuration, which occurs when ${\tau }_{{\rm{com}}}/{\tau }_{1a}\ll 1$. This ratio takes the form:

10 $$\frac{{\tau }_{{\rm{com}}}}{{\tau }_{1a}}=9\frac{{N}_{f}{\varepsilon }^{2}{\rm{\Delta }}{a}^{2}}{{\rm{\Delta }}{N}^{2}{b}^{4}}{e}^{-3{\rm{\Delta }}{a}^{2}\mathrm{/2}{\rm{\Delta }}N{b}^{2}}\mathrm{.}$$

Assuming only a single-step zipper binding for simplicity, for the *m*th binding event, the remaining *N*_*f*_ = (*M* − *m* + 1)Δ*N monomers remain free*. Remembering that the total number of monomers, *N* = *M*Δ*N*, and that the *M*th receptor is placed at a distance *a* = *M*Δ*a*, this condition takes the form:

11 $$m\gg M+1-\frac{MN{b}^{4}}{9{\varepsilon }^{2}{a}^{2}}{e}^{3{a}^{2}\mathrm{/2}MN{b}^{2}}\mathrm{.}$$

We can define the crossover point, ${m}_{{\rm{com}}}^{\ast }$, at which the binding time becomes comparable to the characteristic time of the center of mass diffusion by demanding equality in Eq. (11). So the equilibrium theory of binding to a distant site is valid at $m\gg {m}_{{\rm{com}}}^{\ast }$, and in the opposite limit the dynamics is determined by non-equilibrium (non-Markovian) statistics.

How does this affect the zipper action? Let us assume that the polymer chain is initially equilibrated, with its center of mass close to the current point of grafting. After reaching for the next receptor site, the chain binds there, and then the remaining free chain finds its center of mass a distance Δ*a* out of equilibrium. The entropic force due to this stretching of the chain will provide an impetus to move the center of mass of the remaining chain to re-equilibrate above the new grafting position, and we can write down the dynamical equation for the movement of the center of mass:

12 $$-({N}_{f}\gamma )\dot{x}-\frac{3{k}_{B}T}{2{N}_{f}{b}^{2}}(x-{\rm{\Delta }}a)=\mathrm{0,}$$

where, again, *N*_*f*_ is the number of monomers in the remaining free chain. It follows that the relaxation time to the new equilibrium of the free chain is given by

13 $${\tau }_{{\rm{drift}}}=\frac{2{N}_{f}^{2}{b}^{2}}{3D}\mathrm{.}$$

Then, following a similar method to before, we can find a condition on *m* for the drift time to be dominant, ${\tau }_{{\rm{drift}}}/{\tau }_{1a}\gg 1$:

14 $$m\ll M+1-\frac{1}{\sqrt{6}}\frac{b}{\varepsilon }{e}^{3{a}^{2}\mathrm{/4}MN{b}^{2}},$$

because for early binding events (small *m*), there is a lot of free chain, and so *τ*_drift_ is large. For later binding events, the remaining free chain is able to equilibrate fast, and so there is no need to account for the drift of center of remaining mass.

We can define the other crossover point, ${m}_{{\rm{drift}}}^{\ast }$, at which the chain binding changes from being limited by the chain relaxation time, given by Eq. (13), to being limited by the time *τ*_1*a*_ to reach the next binding site, by setting equality in (14). It turns out that, in spite of subtle differences, the crossover expressions are quite close numerically: ${m}_{{\rm{com}}}^{\ast }\approx {m}_{{\rm{drift}}}^{\ast }={m}^{\ast }$. So when the equilibrium expression for the binding time is valid ($m\gg {m}_{{\rm{com}}}^{\ast }$) it is also the case that the total binding time is dominated by the reaching time. On the other hand, when the chain is not equilibrating fast enough ($m\ll {m}_{{\rm{com}}}^{\ast }$), it is also the case that the binding is limited by the slow drift of the chain center of mass.

Therefore, the first *m* ^*^ binding events (when the free chain segment is still long) will be relaxation-limited, while the last (*M* − *m* ^*^) events are independent of the chain length. The effective binding time takes the form:

15 $$\tau =\sum _{m=1}^{{m}^{\ast }}{\tau }_{{\rm{drift}}}(m)+(M-{m}^{\ast }){\tau }_{{\rm{1a}}}$$

(neglecting the weaker effect of accelerated zipper, for clarity). If *m* ^*^ < 1, which is always the case at small *M*, then all binding events are reach-limited, and the expression returns to the simple linear zipper *τ* = *Mτ*_1a_.

**Figure 4:**
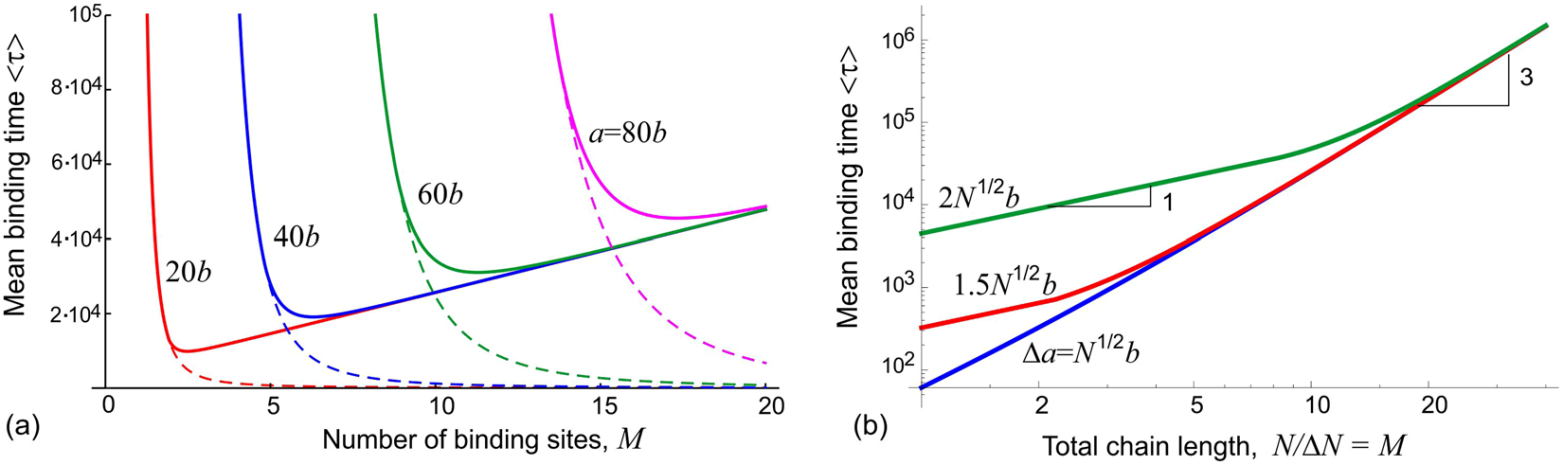
The adhesion time is plotted in units of *b* ^2^/*D*, for *ε* = *b*. (**a**) The linear plot for a fixed *N* = 100. The set-up is *M* receptors, with the final receptor placed at varying distances *a* away in different curves (labelled on the plot). The dashed line shows how the reaction-limited time reduces as *M* increases. (**b**) The log-log plot, for fixed Δ*N* = *N*/*M* = 10. From top to bottom, the distance between consecutive receptors ${\rm{\Delta }}a=\sqrt{{\rm{\Delta }}N{b}^{2}}$, $1.5\sqrt{{\rm{\Delta }}N{b}^{2}}$, and $2\sqrt{{\rm{\Delta }}N{b}^{2}}$. For shorter chains, there is a reach-limited linear ‘zipper’ region (gradient slope of 1 is shown), before switching to the cubic increase of time with length as chains get longer (gradient slope of 3 is shown), see Eq. (18). For larger Δ*a*, the linear region is extended.

Figure 4(a) illustrates how increasing the number of intermediary binding sites affects the total time to adsorb a chain, which is essentially the time to bind to the final receptor a fixed distance away. This is analogous to Fig. 3(a) obtained in the fully equilibrium-chain setting; in fact, the dashed lines in Fig. 4(a) give the lines of Eq. (8) as in Fig. 3(a). We see that for small *M*, increasing the number of binding sites along the chain reduces the total binding time, because the process is purely reach-limited (*m* ^*^ < 0)). As the number of intermediary sites increases, however, we cross into a regime limited by the relaxation (drift) of the chain center of mass. Here, increasing the number of sites lowers the drift force in the Langevin equation, and so the chain will actually take longer to reach the terminal receptor. Note that all curves saturate on the same line, because the relaxation-limited time does not depend on *a*: making the summation in Eq. (15) for *m* ^*^ = *M* gives a linear estimate *τ* ≈ *M*(2*Nb* ^2^/9*D*) for this section of the curves in Fig. 4(a).

Between these two effects: the zipper time, which becomes shorter when more receptors are added on a fixed interval, and the relaxation (drift) time of the chain center of mass, there is clearly an optimal number of binding sites that achieves the shortest time for the complete chain adhesion. To find this point, one has to solve the derivative of Eq. (15): *dτ*(*M*)/*dM* = 0. This is a complicated algebraic task, which simplifies in the limit *a* ^2^ > *Nb* ^2^, that is, when the chain needs to absorb by stretching over some distance. In this case the minimum of the adhesion time is for the *M* determined by the equation:

16 $$16N{\varepsilon }^{3}{M}^{4}-2\sqrt{6}{a}^{2}b{e}^{\frac{9{a}^{2}}{4MN{b}^{2}}}=0.$$

This transcendent equation has a solution in terms of the Lambert W-function, or the product logarithm, but this is too cumbersome to get a clear understanding. However, in the limit ${a}^{2}\gg N{b}^{2}$, the leading contribution to the ‘optimal’ number of receptors for the shortest adhesion time takes the form

17 $${M}_{{\rm{o}}{\rm{p}}{\rm{t}}}\approx \frac{3{a}^{2}}{4N{b}^{2}}{[{\rm{l}}{\rm{n}}(\frac{9{a}^{2}}{16N{b}^{2}}\frac{\varepsilon }{b}\sqrt{\frac{3}{2}})]}^{-1}=\frac{3{a}^{2}/4{R}_{g}^{2}}{{\rm{l}}{\rm{n}}(0.69[{a}^{2}/{R}_{g}^{2}](\varepsilon /b))}.$$

In Fig. 4(b) we instead fix the distance between receptors, Δ*a*, and the chain length between binding sites, Δ*N*, as in Fig. 3(b). When we plot the total adhesion time on a log-log scale, one can clearly see the two distinct regimes: the reach-limited at small *M* (the linear zipper increase), changing to the cubic increase as we go to larger *M* and the relaxation-limited regime. In this latter regime, Eq. (13) with *N*_*f*_ = [*M* − *m* + 1]Δ*N* dominates the contributions to the total adhesion time (15), producing the dominant cubic dependence on the number of receptors, as illustrated in the plot:

18 $$\tau \approx \sum _{m=1}^{M}\frac{\mathrm{2(}N-[m-\mathrm{1]}{\rm{\Delta }}N{)}^{2}{b}^{2}}{3D}\to \frac{2{M}^{3}{\rm{\Delta }}{N}^{2}{b}^{2}}{9D}+\mathrm{...}$$

## Conclusions

The biggest limitation of our model is the specificity of all receptors, and their matching binding ligands on the polymer chain. Non-specific binding of chain segments to the surface has already been covered for uniform surfaces^6–10^, and the case of spaced receptors is relevant for applications such as receptor targeting with multivalent polymers^34^. Could this be considered within the framework of our model? Here, in order to have the dominance of the zipper pathway, we need to be assured that there is no competition for the same receptor by different binding ligands (and therefore a possibility of mis-assembly). This occurs when the second (or higher number *q*) ligand along the chain takes a longer time to bind to the nearest receptor. The binding time for forming a loop of (*qN/M*) monomers, binding a distance Δ*a*, is *τ*_1*a*_(*q*) = *q* ^2^exp[(1/*q* − 1)3*M*Δ*a* ^2^/2*Nb* ^2^] ⋅ *τ*_1*a*_, compare with Eq. (3). So the non-specific binding will follow the zipper pathway when $(N/M){b}^{2}\gg \mathrm{(3(1}-\mathrm{1/}q\mathrm{)/4}\,\mathrm{ln}\,q){a}^{2}$, for all *q*. Since this is a decreasing function with *q*, we can replace the function with the value for *q* = 2, and get that $N{b}^{2}\gg 0.54{a}^{2}$ (i.e. when receptors are relatively closely spaced). The full model will apply to non-specific binding in this case, but may have to account for larger loop formation to get accurate predictions. We note the caveat that with larger loops, there is a greater chance of binding to the ‘wrong’ receptor, since the fractional change in free chain length between binding sites decreases.

In the problem of chain adhesion to a sequence of binding sites on a surface, we find that the addition of intermediary binding sites along a chain length has a dramatic ‘zipper effect’, massively decreasing the time for the chain to bind fully along its length. When we examine our main result–the expression for the average binding time in Eq. (8), it is important that for large Δ*a* and large *M*, the dominant binding process is the single-step ‘zipper’ pathway with the mean time approximately equal to *Mτ*_1*a*_, as we can see in the inset of Fig. 3(b). Only occasionally will a chain bind with a double step, and this correction to the binding time also scales linearly with *M*, see Fig. 3(b). It is not until receptors are very tightly grouped (small Δ*a*) that double-step processes start to become relevant. This is suggestive of reality–if a polymer chain has a specific substrate structure to bind to, then steric effects may well force the polymer to bind in a very conserved and controlled sequence (as in nature), just by virtue of the high entropic penalty for binding ‘out of order’.

The existence of an optimal number of intermediate receptors, *M*_opt_ for the shortest time of full chain adhesion, is our second main result. This is an interesting feature, perhaps contrary to an expectation that by reducing the reaction barrier (and the associated individual binding time) one would increase the overall rate of adsorption. For chains with many intermediate receptors, although there is fast attachment to each individual site, the process of moving the center of mass of the remaining free chain down the line of receptors is slow, because the entropic pulling force causing this drift is weak. Conversely, if there are only a few receptors, even though any attachment event will provide a strong force to move the free chain to its new equilibrium position, the binding time itself is prohibitively slow.

The knowledge of these principles of adsorption kinetics are useful in understanding protein self-assembly, e.g. in *β*-sheets at the growing end of an amyloid fibril. In the design of specifically-binding chains, aiming to optimize the rate of self-assembly, we could use this analysis to guide the structural features. This work also shows that careful positioning of receptors relative to each other allows tight control of the order of assembly.
